# Copy of a Letter to Vice Admiral Lord Nelson, from Dr. Gillespie, Physician to the Fleet, Dated August 14, 1805, Victory, at Sea

**Published:** 1805-11-01

**Authors:** Leonard Gillespie

**Affiliations:** Physician to the Fleet


*Copy of a Letter to Vice Admiral Lord Nelson, from Dr. Gillespie, Physician to the Fleet, dated August* 14, 1805, *Victory, at Sea.*

MY LORD,

FROM the shores of the Nile to the gulph of Finland, the fame of your victories has been proclaimed; a nation, grateful for the important services you have rendered her, regards them with admiration; and the page of history has recorded them with eclat.

To me, as physician to the fleet under your Lordship's command, command, at the approaching close of the campaign, belongs the more humble yet useful and grateful task, to lay before your Lordship a concise statement of the state of health in the fleet during the two years campaign in the Mediterranean and West Indies, taken from the weekly reports made to your Lordship by my predecessor, Doctor Snipe, and by myself: together with a few remarks, contained in the enclosed Statement on the causes of the healthy state in which the men under your Lordship's command have been preserved during that period.

If the Roman people, my Lord, decreed a civic crown to the citizen who saved the life of another citizen, how well do the honours which deservedly adorn you as a victorious commander become you, as the humane citizen, and the preserver of the gallant seamen, who, under the command of yourself, have atchieved such wonders, by an unremitting attention to the furnishing the men under your command with every necessary refreshment and comfort, and by a steady and humane system of active discipline, as is demonstrated by the enclosed statement.

I have the honour to be, &c.

LEONARD GILLESPIE,
Physician to the Fleet.

To the Right Hon. Vice Admiral
Lord Viscount Nelson, Commander in Chief, &c. &c. &c.

---

*Abstract of the Weekly Returns made by the Physician to the Fleet under the Command of the Right Hon. Vice Admiral Lord Viscount Nelson, Duke of Bronte, Commander in Chief in the Mediterranean Station, &c. &c. between the* 13*th of August,* 1803, *and the* 4*th of August,* 1805, *during which Time the said Fleet generally consisted of Ten or Twelve Ships of the Line, and Two or Three Frigates, manned by from* 6000 *to* 8000 *Seamen and Marines.*

1803. From August 13 to the end of the year.

| | |
|---|---|
| Number of men deceased on board - | 18 |
| Number sent to the hospitals - - - | 19 |
| Medium number of men on the sick lists - | 185 |
| 1804. Number of deceased on board - | 43 |
| Number sent to the hospitals - - | 46 |
| Medium number of men on the sick lists - | 190 |
| 1805 to August the 4th. | |
| Number of deceased on board - - | 49 |

Number

| | | |
|---|---|---|
| Number sent to the hospitals | - - | 76 |
| Medium number of men on the sick lists | - | 200 |
| Total number of deaths on board | - | 100 |
| Total number sent to the hospitals | - | 141 |
| Medium number of men on the sick lists | | 190 |

Or eighteen to each ship nearly.

The above statement exhibits the most convincing and satisfactory proofs of the advantages arising from the practice of the improvements adopted in this fleet for the purpose of preserving the crews in good health, and the ships wholesome; and if compared with the accounts of the state of health of fleets or squadrons on foreign stations in former wars, the result will be found to shew the importance of the regulations now used in preserving the health and lives of British seamen.

Thus we find Dr. Blane, physician to the fleet in the West Indies, in the year 1781, in a memorial presented by him, in October of that year, to the Lords of the Admiralty, on the health of seamen, deploring the rapid expenditure of seamen in the navy, and stating, that during one year, in a fleet of twenty sail of the line, manned by 12000 seamen, there died on board 715, and in hospitals 862 men, forming a total of 1577 men, of which number only fifty-nine died in consequence of wounds; during the same period 350 men were invalided: the above shews that one man in seven died in the course of one year in the said fleet.

The following causes may be assigned for the high state of health, in which the fleet under the command of Lord Nelson has been preserved for upwards of two years, unexampled perhaps in any fleet or squadron heretofore employed on a foreign station.

1st. To the attention paid by his Lordship to the victualling and purveying for the fleet, in causing good wholesome wine to be used in room of spirits; fresh beef as often as it could possibly be procured; vegetables and fruit were always provided in a sufficient quantity, when they could be purchased, and an abundant supply of excellent sweet water was always allowed to the ship's company.

2dly. The ships were preserved, as far as possible, from the baneful effects of humidity, by avoiding the wetting the decks (at least between the decks), by the use of stoves and ventilators below.

3dly. The constant activity and motion in which the fleet was preserved, being always at sea, and never exposed posed to the consequences of the idleness and intemperance which too often take place on board of ships lying in harbour, may doubtless be assigned as a principal cause of the good state of health of the crews of this fleet.

4thly. Intemperance and skulking were never so little practised in any fleet as in this, as ships were rarely or never in port; the opportunity of procuring spirits, or going to an hospital, by imposing on the surgeon, were difficult or impossible, hence these causes of disease were subtracted.

5thly. Cheerfulness amongst the men was promoted by music and dancing, and theatrical amusements, the example of which was given by the commander in chief in the Victory, and may with reason be reckoned amongst the causes of the preservation of the health of the men.

6thly. The sick were in general very comfortably accommodated, lodged in airy sick births, in many ships placed on a regular sick diet, and supplied with live stock, vegetables, fruit, soft bread, macaroni, and other articles of diet and refreshments, whenever the circumstances of the service, and the situation of the fleet would admit of these supplies being furnished.

7thly. By a standing order of the Commander in Chief, Peruvian bark, mixed in wine or spirits, was regularly served to the men employed in the wooding and watering service; a drachm of Peruvian bark to one gill of spirits or two of wine, was the proportion allowed for each man; to be administered in divided portions, on going on shore, and on returning on board.

The method followed was to give the bark in a small quantity of wine or spirits, and to wash it down with a glass of wine or spirits, mixed with an equal proportion of water; it was found that the spirits answered better as a vehicle for the bark than the wine, as was experienced on board of some of the ships, in which wine had been used, but afterwards left off, and spirits were used in lieu thereof.

By the returns made by the respective surgeons of the ships to the physician to the fleet, reporting on the efficacy of this mode of prevention of the fevers, which might have been occasioned in consequence of the fatigue and exposure to the weather, and immersion in water by the seamen in wooding and watering; it fully appears that the practice entirely obviated every ill effect which might have been occasioned with regard to the health of the wooding wooding and watering parties, and that it effectually prevented the occurrence of fevers, whether intermittent or continued.

This is the more worthy of remark, as it is well known to experienced officers in the navy, that on foreign stations, sickness very often finds its entrance into ships of war, from the wooding and watering parties being first attacked by fevers, in consequence of fatigue and exposure, which fevers often spread amongst the ship's company, and become a formidable and epidemic disease.

LEONARD GILLESPIE,
Physician to the Fleet.

To Lord Nelson,
*Victory, at Sea, Aug.* 14, 1805.

